# Chaperonopathies: Spotlight on Hereditary Motor Neuropathies

**DOI:** 10.3389/fmolb.2016.00081

**Published:** 2016-12-14

**Authors:** Vincenzo Lupo, Carmen Aguado, Erwin Knecht, Carmen Espinós

**Affiliations:** ^1^Molecular Basis of Human Diseases Program, Centro de Investigación Príncipe FelipeValencia, Spain; ^2^INCLIVA & IIS La Fe Rare Diseases Joint UnitsValencia, Spain; ^3^Centro de Investigación Biomédica en RedValencia, Spain

**Keywords:** Distal hereditary motor neuropathy, distal spinal muscular atrophy, DNAJB2, HSPB1, HSPB3, Chaperone, Heat shock protein

## Abstract

Distal hereditary motor neuropathies (dHMN) are a group of rare hereditary neuromuscular disorders characterized by an atrophy that affects peroneal muscles in the absence of sensory symptoms. To date, 23 genes are thought to be responsible for dHMN, four of which encode chaperones: *DNAJB2*, which encodes a member of the HSP40/DNAJ co-chaperone family; and *HSPB1, HSPB3*, and *HSPB8*, encoding three members of the small heat shock protein family. While around 30 different mutations in *HSPB1* have been identified, the remaining three genes are altered in many fewer cases. Indeed, a mutation of *HSPB3* has only been described in one case, whereas a few cases have been reported carrying mutations in *DNAJB2* and *HSPB8*, most of them caused by a founder c.352+1G>A mutation in *DNAJB2* and by mutations affecting the K141 residue in the HSPB8 chaperone. Hence, their rare occurrence makes it difficult to understand the pathological mechanisms driven by such mutations in this neuropathy. Chaperones can assemble into multi-chaperone complexes that form an integrated chaperone network within the cell. Such complexes fulfill relevant roles in a variety of processes, such as the correct folding of newly synthesized proteins, in which chaperones escort them to precise cellular locations, and as a response to protein misfolding, which includes the degradation of proteins that fail to refold properly. Despite this range of functions, mutations in some of these chaperones lead to diseases with a similar clinical profile, suggesting common pathways. This review provides an overview of the genetics of those dHMNs that share a common disease mechanism and that are caused by mutations in four genes encoding chaperones: *DNAJB2, HSPB1, HSPB3*, and *HSPB8*.

## Chaperones and chaperonopathies

Chaperones (Hartl et al., 2011; Smith et al., 2015) are proteins that, together with the protein degradation machinery (proteasomes, macroautophagy, etc.), contribute to the quality control apparatus and to the proteostasis of a cell. Typically, chaperones recognize other proteins (usually called their clients) to assist in their folding so that they attain their functional conformation at the sites where they must act. Most chaperones are promiscuous and they bind to many clients, although others (dedicated chaperones) restrict their associations to one or a few proteins. However, the information available on the molecules that interact with specific chaperones is still incomplete.

Chaperones also participate in other important processes, such as: (i) the reversion of erroneous folding of newly synthesized proteins; (ii) the prevention of the formation of improper protein aggregates and their disassembly; (iii) the escorting of proteins to their functional sites, including translocation across membranes and the assembly of functional protein-protein, protein-DNA or protein-RNA complexes; and (iv) the sequestering of proteins that are damaged or unable to fold properly to the intracellular protein degradation machinery for destruction. Most of these processes require energy and, therefore, some chaperones have ATP-binding sites and ATPase activity (e.g., Hsp90, Hsp70). By contrast, ATP-independent chaperones must cooperate with the former to carry out such functions. In fact, chaperones tend to assemble into synergistic multi-chaperone complexes of distinct sizes, containing chaperones from the same or different families, as well as other proteins that assist them in their functions, thereby forming an integrated chaperone network in the cell.

Chaperones can either be constitutively expressed, induced by stress (usually but not exclusively, heat shock) or both. Most chaperones induced by heat shock are frequently called heat shock proteins (HSPs). Chaperones, including HSPs, are sometimes classified into six major families according to their molecular mass, although a gross distinction is made between the larger (e.g., the Hsp100, Hsp90, Hsp70, Hsp60, and Hsp40 co-chaperones) and smaller (sHsp, 12–43 kDa, although the vast majority are 30 kDa or less) chaperones. Each group comprises various chaperones and in the human genome, for example, 10 different chaperones have been identified in the sHsp family (HspB1-HspB10). Thus, and although the total number of chaperones in humans is still expanding, an up to date and conservative estimate of their total number would be about 100 genes (Kakkar et al., 2014). Of course, these genes give rise to a much larger number of proteins due to the different transcriptional, translational and post-translational events and modifications they are subjected to. Given the range of activities undertaken by chaperones and the vast number of multimeric complexes that they form with other chaperones, some functional redundancies are likely to exist in their extended networks. Therefore, a single chaperone, or even of a group of dedicated chaperones, would not be expected to be exclusively responsible for a specific task with a particular client, and defects in one chaperone can usually be compensated by others, albeit more or less successfully. Together with the possible lethality associated with the loss of some important chaperones, this redundancy might explain the relatively low number of diseases known to be produced by mutations in genes encoding chaperones (Macario and Conway de Macario, 2007; Kakkar et al., 2014).

## Proteopathies and chaperonopathies

There are many disorders, some that are well known, in which specific misfolded proteins aggregate and accumulate in cells (Walker et al., 2006). Classical examples are Huntington's, Parkinson's and Alzheimer's diseases, although they are not primarily due to defects in the machinery that assist proteins to fold properly but rather, to defects in the specific proteins that accumulate in each disease (e.g., huntingtin, alpha-synuclein, amyloid-beta peptide, and tau). Therefore, these diseases can be referred to as proteopathies or proteinopathies and in principle, they are not considered to be chaperonopathies.

Nevertheless, genetic or post-transcriptional defects in chaperones may be pathological given their role in protein folding. In fact, and despite the potential functional redundancy of chaperones, mutations in genes encoding these proteins have been associated with various disorders that can be collectively referred to as chaperonopathies (Macario and Conway de Macario, 2007). These mutations can affect different yet important domains of a chaperone (e.g., the ATP binding site, client recognition site, sites for interaction with other chaperones, etc.), but they can also affect other sites regulating the expression or the activity of the chaperone. The role of chaperones implies that chaperonopathies may be associated with the aggregation of misfolded proteins but, as mentioned above, such diseases differ from proteinopathies with respect to the protein that is altered (either chaperones or other proteins).

## The growing list of chaperones involved in distal hereditary motor neuropathies

Distal hereditary motor neuropathies (dHMN) or distal spinal muscular atrophies (dSMA) are a group of rare hereditary neuromuscular disorders characterized by an atrophy that affects peroneal muscles in the absence of sensory symptoms (Harding, 1993). Classically, patients experience progressive distal weakness and atrophy affecting the lower limbs, which subsequently spreads to the proximal muscles and ultimately reaches the upper limbs as the disease progresses, with the possible appearance of foot deformities. Other additional manifestations include ataxia or pyramidal tract signs, although these are unusual. These symptoms contrast with those of Charcot-Marie-Tooth disease (CMT) or hereditary motor sensory neuropathy (HMSN), conditions in which sensory involvement is also evident. However, there are some forms of CMT, in particular in axonal CMT or CMT type 2 (CMT2), in which only minor sensory involvement is recognized, and it is difficult to distinguish dHMN from CMT2 (Harding and Thomas, 1980). In fact, some genetic overlap is observed in CMT and dHMN as both conditions can be caused by mutations in the same gene, and even by the same mutation.

To date 23 genes associated with dHMN have been reported (Neuromuscular Disease Center, http://neuromuscular.wustl.edu/synmot.html), although no molecular diagnosis is available in most dHMN patients (Rossor et al., 2012a). Distinct activities are affected in motor-nerve disease, including: protein folding/misfolding (HSPB1, HSPB3, HSPB8, DNAJB2, and BSCL2), RNA metabolism (IGHMBP2, SETX, and GARS), axonal transport (DYNC1H1, DCTN1), cation channel activity (ATP7A, TRPV4), transcriptional control (FBXO38), etc. Here we will focus exclusively on dHMNs that involve mutations in the chaperone genes *HSPB1, HSPB8, DNAJB2*, and *HSPB3*, all four encoding ATP-independent chaperones. Although, compensatory mechanisms driven by the relationships and redundancies within the chaperome can overcome specific chaperone defects, this does not appear to be the case here, as in other diseases. Indeed, even when this compensation occurs, the chaperone activity associated to the defective chaperones would be modified considerably.

The ***DNAJB2/HSJ1*** gene is a member of the HSP40/DNAJ co-chaperone family, characterized by a highly conserved domain of about 70 amino acids, the J domain. This domain allows proteins of this family to interact with Hsp70, and to regulate its ATPase-dependent activity in protein folding and in protein complex dissociation (Hageman et al., 2010). Moreover, spliced transcript variants have been described for the *DNAJB2* gene that encode different isoforms, one of which, DNAJB2a, participates in the resolution of protein aggregates associated with important neurodegenerative diseases (Chen et al., 2016 and references cited therein). Although this protein is mainly expressed in the brain, it has also been localized in normal and diseased skeletal muscle, where it is thought to influence protein turnover through the ubiquitin-proteasome pathway (Claeys et al., 2010). DNAJB2 interacts with ubiquitin chains and their fusion proteins, and since the proteasome mediates the degradation of selected proteins, it is possible that some of these proteins are related to the cytoskeleton (microtubules, intermediate filaments, and microfilaments), in accordance with the role of the other chaperones involved in dHMN (see below).

There are 10 cases where autosomal recessive inheritance has been associated to mutations in the *DNAJB2* gene (Table 1). The first mutation was reported in homozygosis, *DNAJB2* c.352+1G>A, and it was identified in a Moroccan family with a dHMN phenotype (dHMN5) by genome wide mapping (Blumen et al., 2012). In this case, the expression of DNAJB2 was dampened in fibroblasts from the patients and overexpression of the protein reduced the formation of inclusions in a neuronal cellular model, suggesting DNAJB2 is active in motor neurons and/or muscle (Blumen et al., 2012). Two additional homozygous mutations were later described in the *DNAJB2* gene, c.229+1G>A and c.14A>G (p.Y5C), in a family diagnosed with dHMN (dHMN5) and another with CMT2 (CMT2T), respectively (Gess et al., 2014). More recently, a homozygous large deletion was reported in a family with spinal muscular atrophy and parkinsonism, broadening the clinical spectrum of *DNAJB2* related neuropathies (Sanchez et al., 2016).

**Table 1:** **Mutations reported in ***DNAJB2, HSPB1, HSPB3*** and ***HSPB8*** involved in hereditary neuropathies**.

**Gene**	**HGVS (nucleotide)**	**HGVS (protein)**	**Disease/Phenotype**	**References**
*DNAJB2*	c.352+1G>A	donor site	dHMN/CMT2	Blumen et al., 2012; Frasquet et al., 2016; Lupo et al., 2016
	c.229+1G>A	donor site	dHMN	Gess et al., 2014
	c.14A>G	p.Y5C	dHMN	Gess et al., 2014
*HSPB1*	c.20C>G	p.P7R	CMT2	Luigetti et al., 2010
	c.45C>A	p.S15R	Peripheral neuropathy	Antoniadi et al., 2015
	c.100G>A	p.G34R	HMSN	Capponi et al., 2011; Muranova et al., 2015
	c.116C>T	p.P39L	dHMN/CMT2	Houlden et al., 2008; Muranova et al., 2015; Yavarna et al., 2015
	c.121G>A	p.E41K	dHMN	Capponi et al., 2011; Muranova et al., 2015
	c.250G>A	p.G84R	CMT2	Manganelli et al., 2014
	c.250G>C	p.G84R	dHMN	James et al., 2008; Fischer et al., 2012; Nefedova et al., 2015
	c.257C>T	p.S86L	dHMN/ALS	Scarlato et al., 2015
	c.295C>A	p.L99M	dHMN/CMT2	Houlden et al., 2008; Nefedova et al., 2015
	c.380G>T	p.R127L	CMT2	Hoyer et al., 2014; Ylikallio et al., 2015
	c.379C>T	p.R127W	dHMN	Evgrafov et al., 2004; Almeida-Souza et al., 2011
	c.404C>G	p.S135C	CMT2	Benedetti et al., 2010; Oberstadt et al., 2016
	c.404C>G	p.S135C	CMT2	Benedetti et al., 2010; Oberstadt et al., 2016
	c.404C>T	p.S135F	CMT2	Evgrafov et al., 2004; Almeida-Souza et al., 2010, 2011
	c.404C>A	p.S135Y	CMT2	Ylikallio et al., 2014
	c.407G>T	p.R136L	dHMN/CMT2	Capponi et al., 2011; Gaeta et al., 2012; Stancanelli et al., 2015
	c.406C>T	p.R136W	CMT2	Evgrafov et al., 2004; Almeida-Souza et al., 2010, 2011
	c.418C>G	p.R140G	dHMN/CMT2	Houlden et al., 2008; Nefedova et al., 2015
	c.421A>C	p.K141Q	dHMN	Ikeda et al., 2009; Nefedova et al., 2013; Maeda et al., 2014
	c.452C>T	p.T151I	dHMN	Evgrafov et al., 2004; Almeida-Souza et al., 2010, 2011
	c.490A>G	p.T164A	CMT2	Lin et al., 2011
	c.523C>T	p.Q175X	CMT2	Rossor et al., 2012b
	c.539C>T	p.T180I	dHMN/CMT2	Luigetti et al., 2010
	c.545C>T	p.P182L	dHMN	Evgrafov et al., 2004; Almeida-Souza et al., 2010, 2011
	c.544C>T	p.P182S	dHMN	Kijima et al., 2005
	c.562C>T	p.R188W	CMT2	Capponi et al., 2011
	c.365-13C>T	acceptor site	CMT2	Benedetti et al., 2010
	c.-217T>C	regulatory	ALS	Dierick et al., 2007
	c.476_477delCT	p.P159RfsX41	Peripheral neuropathy, early onset	Mandich et al., 2010; Capponi et al., 2011
	c.505delA	p.M169CfsX4	CMT	DiVincenzo et al., 2014
	c.171_172insGCGCCCT	p.L58AfsX105	CMT	DiVincenzo et al., 2014
*HSPB3*	c.21G>T	p.R7S	dHMN	Kolb et al., 2010
*HSPB8*	c.423G>C)	p.L141N	dHMN/CMT2	Irobi et al., 2004
	c.421A>G	p.L141E	dHMN	Irobi et al., 2004
	c.423G>T	p.L141N	CMT2	Tang B. S. et al., 2005
	c.422A>C	p.L141T	CMT2	Nakhro et al., 2013
	c.151insC	p.P173SfsX43	Distal myopathy/dHMN	Ghaoui et al., 2016

*ALS, Amyotrophic lateral sclerosis; CMT2, Charcot-Marie-Tooth disease type 2 or axonal; dHMN, Distal hereditary motor neuropathy; HMSN, hereditary motor and sensory neuropathy*.

To date, the remaining known patients with mutations in the *DNAJB2* gene carry the c.352+1G>A mutation in homozygosis: 5 families from Spain (Frasquet et al., 2016; Lupo et al., 2016) and one from Brazil (Teive et al., 2015). These Spanish families were investigated by haplotype analysis and they carried the same homozygous haplotype. Hence, the *DNAJB2* c.352+1G>A mutation appears to be a founder event (Lupo et al., 2016), and it is shared with a family reported elsewhere (Blumen et al., 2012). The patients in Spain displayed a dHMN or CMT2 phenotype and, in some cases, initial clinical manifestations that were consistent with dHMN and that subsequently evolved to CMT2 (Frasquet et al., 2016). Moreover, the peripheral motor neuropathy recently described in a Brazilian family carrying the *DNAJB2* c.352+1G>A mutation was associated with parkinsonism and cerebellar ataxia (Teive et al., 2015). Some patients show parkinsonian symptoms (Frasquet et al., 2016; Sanchez et al., 2016; Teive et al., 2015), which probably are due to the DNAJB2 mutations. Other additional symptoms such as cerebellar ataxia may be coincidental. Further studies of a larger analytical series will be necessary to define the clinical manifestations associated with *DNAJB2* mutations in more depth.

**HSPB1, HSPB3**, and **HSPB8** are the three other chaperones associated with dHMNs, and they are all members of the sHsp family. These proteins are characterized by a highly conserved α-crystallin domain that is related to their chaperone activity, which is more closely associated with an 80–100 amino acid domain in the C- rather than the N-terminal region of the protein (Nefedova et al., 2015). These chaperones are normally found as monomers, but under stress, they tend to also interact with each other to form large, labile homo- and hetero-oligomeric complexes of more than twenty identical or different subunits, driving their recognition and interaction with new protein clients (Arrigo, 2013). Certain sHsp are tissue specific, while others are more ubiquitously expressed in function of the tissue and conditions. The main role of sHsps is to carry their denatured clients to ATP-dependent chaperones for renaturation or to the cell's protein degradation machinery (proteasomes and autophagosomes). In terms of dHMN and HMSN, sHsps stabilize the activities of the cell cytoskeleton, interacting with most of its proteins components, as well as preventing oxidative stress (Nefedova et al., 2015).

Autosomal dominant mutations in the ***HSPB1/HSP27*** gene were first described in four families with dHMN (dHMN2B) and in one family with CMT2 (CMT2F) (Evgrafov et al., 2004). More than 30 different mutations causing dHMN or CMT2 have since been described in the *HSPB1* gene, some of which also produce other manifestations (Table 1; Evgrafov et al., 2004; Kijima et al., 2005; Tang B. et al., 2005; Chung et al., 2008; Houlden et al., 2008; James et al., 2008; Ikeda et al., 2009; Luigetti et al., 2010; Mandich et al., 2010; Solla et al., 2010; Murphy et al., 2012; Rossor et al., 2012b; Sivera et al., 2013; Ylikallio et al., 2014, 2015). An autosomal recessive mutation in the *HSPB1* gene was identified in a consanguineous family with a similar clinical profile (Houlden et al., 2008). On the whole, *HSPB1* mutations are inherited dominantly and while most involve a change in one codon, they may also produce a frameshift or premature stop codons. The protein encoded by this gene is ubiquitously expressed and it is induced by environmental stress, translocating from the cytoplasm to the nucleus to influence stress resistance and produce other changes. The known mutations are located in all three domains of the protein: N-terminus, α-crystallin and C-terminus. These *HSPB1* mutations mostly modify the oligomeric state of the protein, usually negatively but also positively (certain mutations in the α-crystallin domain), altering its chaperone activity and in both cases affecting normal cytoskeletal function. HSPB1 is involved in the organization of the neurofilament network, which is important to maintain the axonal cytoskeleton and transport, and indeed, overexpression of HSPB1 mutants produces protein aggregates and altered neurofilament transport in the axon (Evgrafov et al., 2004; Ackerley et al., 2006; Zhai et al., 2007). Thus, an increased interaction with tubulin and an enhanced stability of the microtubule network has been observed for some mutants (Almeida-Souza et al., 2011). Moreover, there are severe defects in axon transport in transgenic mice expressing human mutant HSPB1 in neurons due to a decrease in acetylated α-tubulin (d'Ydewalle et al., 2011). As a result, inhibitors of histone deacetylase 6 (HDAC6, a client of HSPB1 that acetylates α-tubulin) have successfully reversed the axonal loss in a mouse model of CMT2F that expresses mutant HSPB1 (d'Ydewalle et al., 2011). HSPB1 is also involved in a variety of human diseases, such as cancer, Alzheimer's disease and heart disease (Sun and MacRae, 2005).

At present, only one family is thought to carry clinical mutations in the ***HSPB3/HSPL27*** gene: a missense mutation c.21G>T (p.R7S) described in two affected sisters who suffer from dHMN (dHMN2C) (Table 1; Kolb et al., 2010). The function of HSPB3 is not fully understood, although replacing the positively charged R7 residue with a neutral polar amino acid would affect its structure and therefore, its proprieties. In contrast to the ubiquitous expression of *HSPB1* and *HSPB8, HSPB3* is more tissue specific (heart, brain, skeletal and smooth muscle) and it is expressed strongly in muscle (Sugiyama et al., 2000). HSPB3 interacts with HSPB2 and these two proteins in turn both interact with HSPB8, potentially contributing to maintain myofibril integrity (Fontaine et al., 2005). Finally, HSPB3 and HSPB2 are upregulated in a mouse model for spinal and bulbar muscular atrophy (SBMA), an inherited motoneuron disease (Rusmini et al., 2015).

Mutations in the ***HSPB8/HSP22*** gene were first associated with dHMN (dHMN2A) (Irobi et al., 2004) and later, with CMT2 (CMT2L) (Table 1; Tang B. S. et al., 2005). Four mutations have been described and they all affect position K141: c.423G>T/c.423G>C (p.K141N), c.421A>G (p.K141E), and c.422A>C (p.K141T). These mutations are all transmitted in an autosomal dominant fashion (Irobi et al., 2004; Tang B. S. et al., 2005; Nakhro et al., 2013), and this hot-spot residue is located in a hydrophobic strand of the α-crystallin domain. The mutations eliminate the positive charge of the K41 amino acid, which will affect the interactions of HSPB8 with other sHsps like HSPB27, HSPB3, and HSPB2 (Irobi et al., 2004; Fontaine et al., 2006; Kasakov et al., 2007; Nakhro et al., 2013). Mutational screening in a large clinical series revealed additional patients but no novel mutations associated with dHMN or CMT2 (Dierick et al., 2008; Sivera et al., 2013; Fridman et al., 2015). However, two mutations, c.421A>G (p.K141E), and c.151insC (p.P173SfsX43) were recently described in two unrelated families with a new distal neuromyopathy phenotype, expanding the clinical phenotype associated with *HSPB8* (Ghaoui et al., 2016).

HSPB8 is ubiquitously expressed (particularly strongly in the spinal cord, and especially in motor and sensory neurons), and it acts as a chaperone and a regulator of apoptosis (Shemetov et al., 2008). HspB8 acts as a chaperone in association with the co-chaperones Bag3 and Stub1, stimulating chaperone-assisted selective macroautophagy in muscle to maintain the actin cytoskeleton (Arndt et al., 2010). Expression of HSPB8 mutants in cell models promotes the formation of intracellular aggregates and it augments cell death (Benn et al., 2002; Irobi et al., 2004). These protein aggregates are also observed in fibroblasts from patients who carry *HSPB8* mutations, and they are coupled to a decrease in mitochondrial membrane potential and a reduction in cell viability (Irobi et al., 2012; Vicario et al., 2014). Although the pathological mechanisms underlying these conditions remain enigmatic, specific motor neuron degeneration is associated with *HSPB8* mutations (Irobi et al., 2010). In addition, expression of this protein can be induced by estrogen in estrogen receptor-positive breast cancer cells, indicating a role in carcinogenesis, and suggesting the possible involvement of HspB8 in regulating cell proliferation and apoptosis.

Since mutations in these four chaperones, as well as those in other genes, produce a similar pathological phenotype, it would seem obvious that they must share some pathogenic pathways. It has been proposed that most, if not all, of the proteins affected in dHMN/CMT2 are related with the impaired axonal trafficking of cell components (Bucci et al., 2012; Gentil and Cooper, 2012). Considering the activity of all the chaperones described above, it appears that mutations in all these genes could affect the cytoskeleton, either by interacting with relevant proteins (e.g., in the case of the sHsps) or by regulating their specific degradation (e.g., in the case of DNAJB2 and HspB8). Since the cytoskeleton participates in axonal transport, as well as in the dynamics of various organelles and plasma membrane receptors, there are clear potential relationships with other mutations that cause dHMN/CMT2. To date there are no effective treatments for these diseases and therefore, much more research is needed to understand the consequences of each specific mutation that provokes them. However, one potential therapy to be considered, at least in certain cases of these chaperonopathies, could be to overexpress the chaperone to rescue its defective functions. Indeed, the overexpression of HspB8 ameliorates the accumulation of aggregates associated with the p.P182L mutation in HspB1 (Carra et al., 2010), or the effects on its clients, as illustrated by the use of inhibitors of histone deacetylase 6 to treat CMT2F (d'Ydewalle et al., 2011).

## Author contributions

Conceptualization: EK, CE; Writing-draft, review and editing: VL, CA, EK, CE; Funding acquisition and supervision: EK, CE.

### Conflict of interest statement

The authors declare that the research was conducted in the absence of any commercial or financial relationships that could be construed as a potential conflict of interest.
